# A Case of Impaired Upper Esophageal Sphincter Opening Due to Sarcopenic Dysphagia Successfully Treated With Balloon Dilatation

**DOI:** 10.7759/cureus.65595

**Published:** 2024-07-28

**Authors:** Keishi Okamoto, Kenjiro Kunieda, Tomohisa Ohno, Akiko Nomoto, Ichiro Fujishima

**Affiliations:** 1 Department of Rehabilitation, Hamamatsu City Rehabilitation Hospital, Shizuoka, JPN; 2 Department of Neurology, Gifu University Graduate School of Medicine, Gifu, JPN; 3 Department of Rehabilitation Medicine, Hamamatsu City Rehabilitation Hospital, Shizuoka, JPN; 4 Department of Dentistry, Hamamatsu City Rehabilitation Hospital, Shizuoka, JPN

**Keywords:** impaired upper esophageal sphincter opening, resistance training of swallowing, nutritional management, balloon dilatation, sarcopenic dysphagia

## Abstract

A case of an 84-year-old man diagnosed with "probable sarcopenic dysphagia" using the sarcopenic dysphagia diagnostic algorithm is presented. The patient demonstrated improved upper esophageal sphincter (UES) passage by the immediate effect of balloon dilatation. He had suffered a myocardial infarction and was unable to eat orally for approximately a month, presenting with sarcopenia and severe dysphagia, as indicated by the Food Intake LEVEL Scale (FILS) score of 1. Videofluoroscopic examination of swallowing study at 67 hospital days revealed impaired UES opening, with food bolus unable to pass through the UES. After confirming the loss of the gag reflex, we performed balloon dilatation, resulting in improved UES passage. With swallowing rehabilitation using balloon dilatation and appropriate nutritional therapy, the patient progressed to full oral intake and achieved FILS score of 8. This case suggests the effectiveness of combined nutritional therapy and swallowing rehabilitation with balloon dilatation in managing sarcopenic dysphagia. In addition, balloon dilatation could be applied for patients with sarcopenic dysphagia presenting impaired UES opening.

## Introduction

Sarcopenic dysphagia is characterized by dysphagia resulting from sarcopenia affecting both the whole body and the swallowing muscles [1-4]. Older adult patients with sarcopenic dysphagia show weakened pharyngeal contractility and impaired upper esophageal sphincter (UES) opening due to decreased suprahyoid muscles, as assessed by high-resolution manometry [5]. In sarcopenic dysphagia, aggressive nutritional therapy aimed at increasing muscle mass should be combined with swallowing rehabilitation including resistance training for pharyngeal contractility and impaired UES opening [6].

The catheter balloon dilatation method is used to improve UES passage by mechanically dilating cricopharyngeal muscle in patients with impaired UES opening [7]. As for the UES function mechanism during swallowing, high-resolution manometry revealed immediate and prolonged UES opening with decreased UES pressure during swallowing as an immediate effect of the balloon dilatation method [8]. The immediate effect of balloon dilatation has been reported in Wallenberg syndrome and several neuromuscular diseases [7,8], but there are no reports of its effectiveness in a patient with sarcopenic dysphagia.

The characteristic finding in sarcopenic dysphagia is residual pharyngeal residues due to decreased pharyngeal contractility and impaired UES opening [1]. In addition to nutritional therapy and swallowing rehabilitation, mechanical dilatation of the UES may improve the pharyngeal passage of bolus. This case report describes a patient with severe sarcopenic dysphagia whose UES passage was improved by balloon dilatation on videofluoroscopic examination of swallowing (VF) and a combination of nutritional therapy and swallowing rehabilitation enabled full oral intake.

## Case presentation

An 84-year-old man was diagnosed with myocardial infarction and aspiration pneumonia. He had no medical history and was independent in activities of daily living (ADL) until experiencing a myocardial infarction six months before. The patient had been on a dysphagia diet but experienced repeated episodes of aspiration pneumonia within a month. He was then managed only with peripheral parenteral nutrition and was admitted to our hospital 35 days after the recurrence of aspiration pneumonia. Physical examination and nutritional assessment revealed a height of 164 cm, body weight of 36.3 kg, body mass index (BMI) of 13.5 kg/m^2^, a serum albumin of 2.8 g/dl, a creatinine of 0.45 mg/dl, and severe malnutrition diagnosed according to the Global Leadership Initiative on Malnutrition (GLIM) criteria [9]. The patient had reduced general endurance and was completely dependent on ADL. The Mann Assessment of Swallowing Ability (MASA) [10] score was 122, indicating severe dysphagia and severe aspiration. The Food Intake LEVEL Scale (FILS) [11] was level 1 (no swallowing training is performed except for oral care). The head computed tomography (CT) and magnetic resonance imaging (MRI) showed mild brain atrophy but no obvious lesion causing dysphagia. The chest CT showed infiltrative shadows in the bilateral lower lung fields, and he required frequent suctioning.

Diagnosis of sarcopenic dysphagia

**Figure 1:**
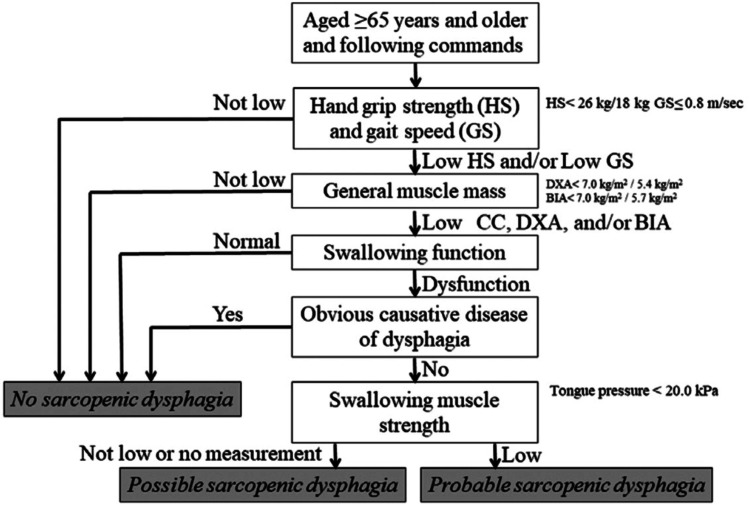
Diagnostic algorithm for sarcopenic dysphagia Diagnostic algorithm used for sarcopenic dysphagia [2]. The patient was classified as a "probable sarcopenic dysphagia." CC, calf circumference; DXA, dual-energy X-ray absorptiometry; BIA, bioimpedance analysis Reference: [2] Mori T, Fujishima I, Wakabayashi H, et al.: Development, reliability, and validity of a diagnostic algorithm for sarcopenic dysphagia. JCSM Clin Reports. 2017, 2:1-10.

The diagnosis of sarcopenic dysphagia was made using the diagnostic algorithm [2] (Figure 1). The patient was classified as "probable sarcopenic dysphagia" based on the assessment of hand grip strength of 7.2 kg on the right and 8.9 kg on the left, unmeasured walking speed, a calf circumference of 24 cm as general muscle mass, no obvious causative disease of dysphagia, and low swallowing muscle strength with a tongue pressure of 16.4 kPa.

Clinical course before the introduction of balloon dilatation

**Figure 2:**
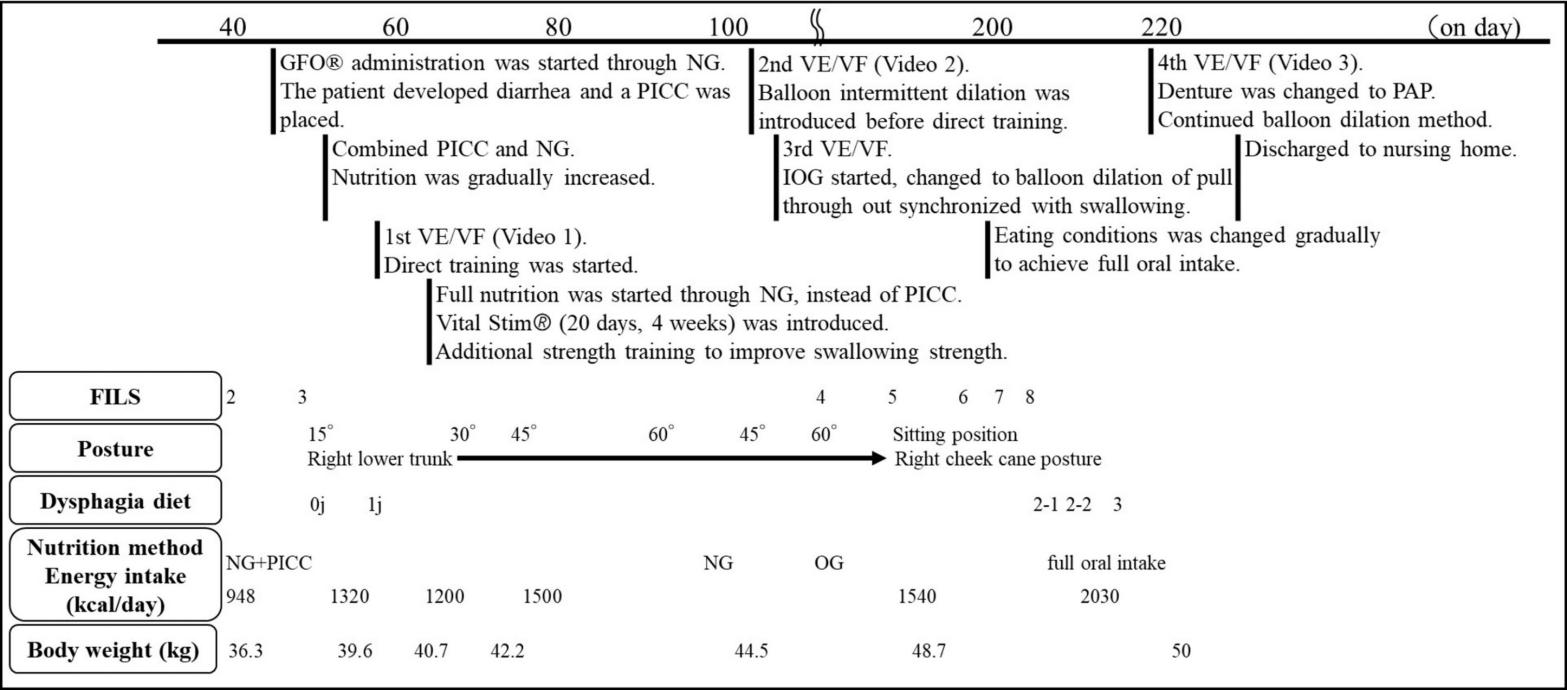
The course of the patient after admission The following graph shows the course of the patient after their admission to our hospital. FILS, Food Intake LEVEL Scale; NG, nasogastric tube feeding; PICC, peripherally inserted central venous catheter; VE, videoendoscopic evaluation of swallowing; VF, videofluoroscopic examination of swallowing; OG, oral gastric feeding; PAP, palatal augmentation prosthesis; j, jelly

The course of the patient after admission is shown in Figure 2. Sarcopenic dysphagia was reported to be treated with high-nutrient energy of ≥30 kcal/ ideal body weight (IBW)/day (kg) [12]. IBW was calculated using the following formula: IBW = height^2 ^(m^2^) × 22 [13]. The patient's IBW was 59.1 kg. The daily energy requirement for the treatment of sarcopenia was calculated as more than 1775 kcal/day. On admission, a nasogastric (NG) tube feeding was started with GFO® (Otsuka Pharmaceutical Factory, Co. Ltd, Tokushima, Japan). However, the patient developed diarrhea, so a peripherally inserted central venous catheter (PICC) was placed. The patient's caloric intake was 948 kcal/day, and the nutrition was gradually increased using a combination of NG tube feeding and parenteral nutrition via PICC. Videoendoscopic evaluation of swallowing (VE) and VF were performed on day 53 to evaluate swallowing function. VE showed no pharyngeal and vocal cord paralysis, but viscous sputum pooling in the epiglottic vallecula and cervical osteophytes on the left side of the posterior pharyngeal wall (Video 1). VF was performed at a 30° reclining posture and showed laryngeal elevation failure, decreased pharyngeal contractility, and impaired UES opening, resulting in the aspiration of pharyngeal residue with jelly (first half of Video 2). Considering the left side of the cervical osteophyte, the patient was placed in a unilateral position with the right lower trunk at 15° to induce the bolus to the right of the pharynx and he could swallow sliced jelly [14] without aspiration (second half of Video 2). Direct therapy with jelly was started on day 54 and was combined with a cough-inducing method using tartaric acid (CiTA) for silent aspiration to expectorate sputum inducing a strong cough [15]. Neuromuscular electrical stimulation (NMES) therapy using VitalStim® (InterReha Co. Ltd, Tokyo, Japan) of the anterior belly of the digastric and geniohyoid muscles was performed for muscle strengthening for four weeks starting on day 66. Forehead exercises for suprahyoid muscles (FESM, "Enge-Odeko-Taiso" in Japanese) were also initiated [16]. On day 74, the patient was transitioned to nutritional therapy with full NG (provided energy of 1320 kcal/day), and the PICC line was removed on day 83.

**Video 1:** First VE of the patient (on day 53) VE showed viscous sputum pooling in the epiglottic vallecula and cervical osteophytes on the left side of the posterior pharyngeal wall (arrowhead). VE, videoendoscopic evaluation of swallowing

**Video 2:** First VF of the patient (on day 53) VF was performed at a 30° reclining posture and showed an inability to elevate the larynx, decreased pharyngeal contractility, and aspiration of the pharyngeal residue with jelly was observed (first half). Considering the left side of the cervical osteophyte, the patient was placed in a unilateral position with the right lower trunk at 15° and he could swallow the jelly without aspiration (second half). VF, videofluoroscopic examination of swallowing

Introduction and subsequent clinical course of balloon dilatation

**Figure 3:**
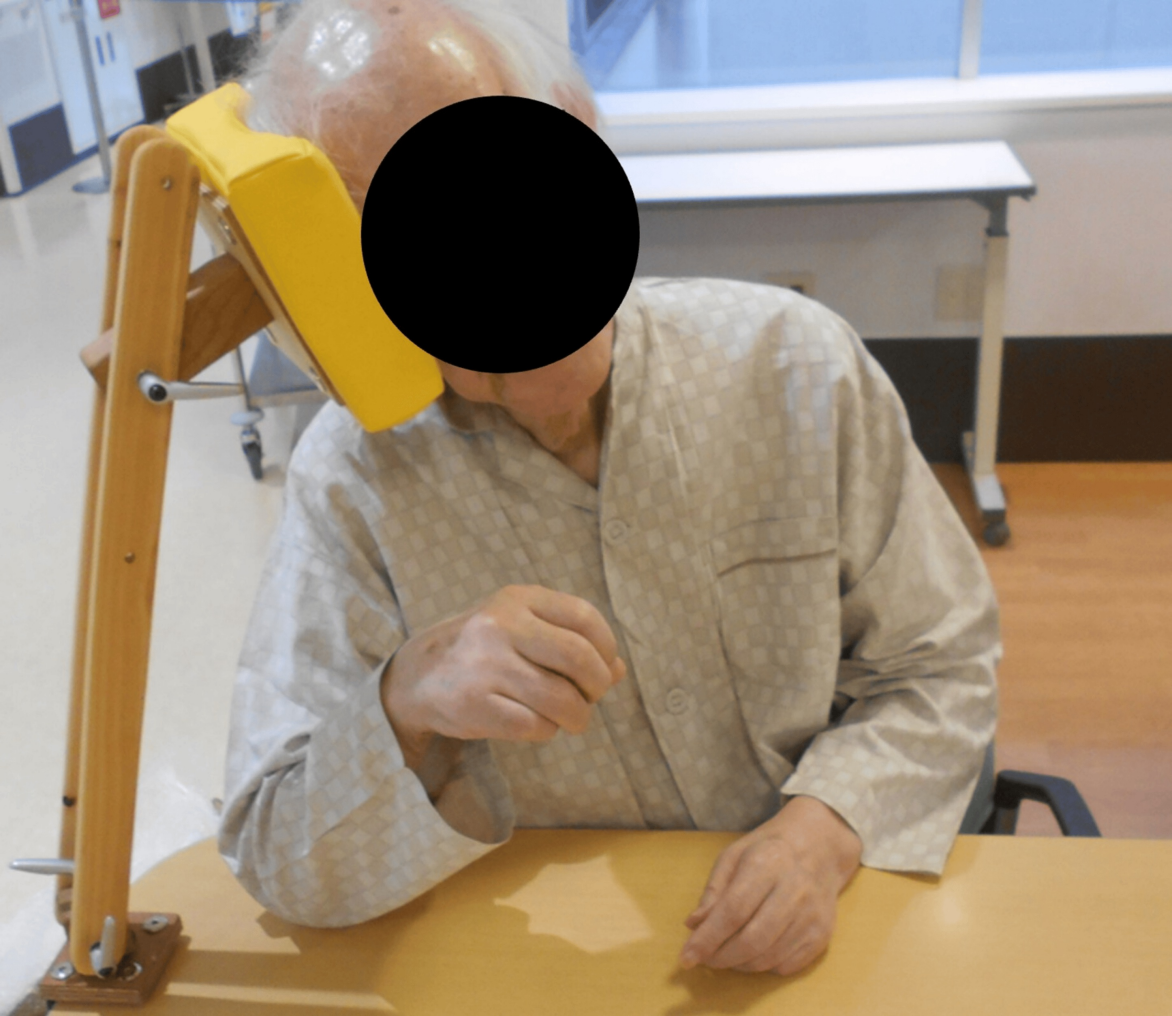
Right cheek cane posture A posture with the trunk and neck tilted to the right in a sitting position and the neck slightly rotated to the left. The Cheek Cane® (Hashimoto Rashi, Japan) is used to establish the correct cheek cane posture. This compensatory posture allows the food mass to be directed to the right UES. UES, upper esophageal sphincter

The second VE/VF was performed on day 102. VF showed residue in the pyriform sinus due to decreased pharyngeal contractility and impaired UES opening. After confirming loss of gag reflex, we performed the intermittent balloon dilatation method [7] using 18 French Biocath® Foley catheter (Medicon Co. Ltd, Osaka, Japan) inflated with 5 mL of air for impaired UES opening. Immediate effect of the balloon dilatation was observed, improving UES passage (Video 3). Therefore, the intermittent balloon dilatation method before direct therapy was introduced on day 103. At the time of the introduction of intermittent oro-gastric tube feeding on day 130, the balloon dilatation method was changed to pull throughout synchronized with swallowing [7]. The fourth VF was performed on day 221, and the balloon dilatation of pull throughout synchronized with swallowing was continued because UES passage had been improving (Video 4). There was also dysfunction of the tongue, so we used a palatal augmentation prosthesis (PAP) [17]. Finally, his oral feeding conditions were improved to three meals a day, in a posture with the trunk and neck tilted to the right in a sitting position and the neck slightly rotated to the left (right cheek cane posture, “migi hozue-enge” in Japanese) (Figure 3), the Japanese Dysphagia Diet of 2021 dysphagia diet 3 (mash easily by the tongue and palate) [18], self-feeding for three meals (FILS 8) with the balloon dilatation of pull throughout synchronized with swallowing before every meal. The provided energy was 2030 kcal/day (34.3 kcal/IBW/day (kg)).

**Video 3:** Second VF of the patient (on day 102) VF showed residue in the pyriform sinus due to decreased pharyngeal contractility and impaired UES opening (first half). The intermittent balloon dilatation method for impaired UES opening (middle) was done. The immediate effect of the balloon dilatation was observed, showing improved UES passage (second half). VF, videofluoroscopic examination of swallowing; UES, upper esophageal sphincter

**Video 4:** Fourth VF of the patient (on day 221) The immediate effect of the balloon dilatation was observed, improving UES passage. The nail-like objects seen on the head and neck are parts of Cheek Cane® (see Figure 3). VF, videofluoroscopic examination of swallowing; UES, upper esophageal sphincter

Physical examination and nutritional assessment revealed an improved nutritional status compared to admission with a body weight of 50 kg, BMI of 18.5, serum albumin of 3.7 g/dl, a creatinine of 0.45 mg/dl, and no applicable to low nutrition diagnosed according to the GLIM criteria. ADL improved to independence, hand grip strength improved to 8.3 kg on the right and 11.7 kg on the left, and tongue pressure improved to 25.6 kPa. The MASA score improved to 162 (dysphagia: moderate, aspiration: mild). The patient was discharged to the nursing home on day 243 and continued balloon dilatation before oral intake.

## Discussion

This is the first report of the effect of balloon dilatation in a patient with sarcopenic dysphagia. In this case report, there are two important findings regarding the treatment of sarcopenic dysphagia. First, balloon dilatation showed an immediate effect in improving UES passage even in cases of sarcopenic dysphagia with incomplete UES opening. Second, combining appropriate nutritional therapy and swallowing rehabilitation with balloon dilatation was important and effective for dysphagia due to sarcopenia.

The most important finding is that balloon dilatation might be effective in improving UES passage in sarcopenic dysphagia. Age-related changes in the swallowing muscles include decreased geniohyoid muscle mass, decreased duration of UES opening, and a compensatory increase in pharyngeal swallowing pressure for impaired UES opening [3-5]. It was reported that sarcopenic dysphagia might exacerbate impaired UES passage when the compensatory increase of pharyngeal swallowing pressure became ineffective [5]. Furthermore, reduced elasticity of the cricopharyngeal muscle and surrounding tissues in this patient with sarcopenic dysphagia may cause contracture and increase residual pressure of UES. The balloon dilatation method is used to improve UES passage by decreasing cricopharyngeal muscle compliance [7]. It was considered that balloon dilatation in patients with sarcopenic dysphagia also might improve UES passage by directly stretching the cricopharyngeal muscle and its surrounding tissues and improving flexibility. It may be important to make sure that there is no gag reflex before performing balloon dilatation. Gag reflex was absent bilaterally in 43% of older adults and in 26% of young subjects [19]. In this case, balloon dilatation was able to be performed due to the absence of the gag reflex.

The second important finding is that appropriate nutritional therapy and swallowing rehabilitation including resistance training are necessary for the effective management of patients with sarcopenic dysphagia. Patients with sarcopenic dysphagia in the recovery phase who received high-nutrient energy of ≥30 kcal/IBW/day (kg) showed significantly higher FILS scores at discharge [12]. In patients with sarcopenic dysphagia, aggressive nutritional therapy to increase muscle mass should be combined with rehabilitation therapy including swallowing exercises [6]. This patient was diagnosed with severe malnutrition according to the GLIM criteria on admission. However, with appropriate and gradual nutritional management, his nutritional status improved. At discharge, he was not applicable to low nutrition diagnosed according to the GLIM criteria, with a final energy intake of 34.3 kcal/IBW/day (kg). In addition, oral feeding training was performed under safe conditions according to the results of VF/VE, as well as resistance training of swallow-related muscles such as NMES and FESM [16] for approximately 200 days. As in previous reports, a combination of appropriate nutritional management and resistance training of the swallowing-related muscles was found to be effective. Strengthening the suprahyoid muscles might facilitate to improve the UES opening. In addition, continuous long-term balloon dilatation might maintain improved UES passage. A previous report showed that it took approximately six months for sarcopenic dysphagia to improve [20]. In this case, managing diarrhea and respiratory infections at the beginning of hospitalization was also a factor in the prolonged hospital stay. Improvement of severe sarcopenic dysphagia might take a long time. As previously reported, prevention is crucial for sarcopenic dysphagia [1].

## Conclusions

This case report highlights the immediate effect of balloon dilatation in improving UES passage in a patient with sarcopenic dysphagia. The combination of appropriate nutritional management and swallowing rehabilitation, including resistance training of swallowing-related muscles and balloon dilatation, might be effective in promoting UES opening. In patients presenting with pharyngeal residue in the pyriform sinus due to sarcopenic dysphagia, balloon dilatation might be a useful therapy.
